# Treatment of children and adolescents with post-traumatic stress in humanitarian crises: systematic review and meta-analysis

**DOI:** 10.2471/BLT.24.292608

**Published:** 2025-05-27

**Authors:** Fiona E Douglas, Chirag Shroff, Richard Meiser-Stedman

**Affiliations:** a Department of Liaison Psychiatry, Cambridge University Hospitals NHS Foundation Trust, Hills Road, Cambridge CB2 0QQ, England.; b Adult Mental Health Services, Cheshire and Wirral NHS Foundation Trust, Chester, England.; c Department of Clinical Psychology & Psychological Therapies, Norwich Medical School, University of East Anglia, Norwich, England.

## Abstract

**Objective:**

To assess the effectiveness of mental health and psychosocial support interventions for addressing post-traumatic stress symptoms in children and adolescents in humanitarian settings, with separate analyses of targeted and universal interventions.

**Methods:**

We systematically searched MEDLINE, Web of Science, PTSDpubs and PsycInfo databases for relevant randomized controlled trials that involved individuals aged 18 years or younger in humanitarian settings and compared psychological interventions with passive controls. Random-effects meta-analyses were performed separately for interventions targeted to specific symptoms and for more general universal interventions. The review was registered on PROSPERO (CRD42023434878).

**Findings:**

The meta-analysis included 16 trials of targeted interventions (*n* = 2356) and 11 of universal interventions (*n* = 3378) that met inclusion criteria. Children and adolescents who received targeted interventions reported significantly fewer post-traumatic stress symptoms after the intervention than controls. These positive effects were sustained at follow-up. Benefits were also observed for depressive symptoms. In addition, universal interventions were associated with significantly fewer post-traumatic stress symptoms. Moderator analyses showed that outcomes were not significantly influenced by country income level, the use of lay or professional therapists, or whether trauma was caused by human-made or natural disasters. However, considerable heterogeneity and a high risk of bias were noted across studies.

**Conclusion:**

Both targeted and universal mental health and psychosocial support interventions were associated with fewer post-traumatic stress symptoms among children and adolescents in humanitarian settings compared with passive controls. As interventions delivered by non-specialists were also successful, task-sharing could be considered where resources are scarce.

## Résumé

**Objectif:**

Évaluer l’efficacité des interventions de santé mentale et de soutien psychosocial pour traiter les symptômes de stress post-traumatique chez les enfants et les adolescents se trouvant dans des situations de crise humanitaire, avec des analyses séparées d’interventions ciblées et universelles.

**Méthodes:**

Nous avons effectué des recherches systématiques dans les bases de données de MEDLINE, de Web of Science, de PTSDpubs et de PsycInfo pour trouver des essais contrôlés randomisés pertinents qui concernaient des personnes âgées de 18 ans ou moins dans des contextes de crise humanitaire et comparaient les interventions psychologiques à un groupe témoin passif. Des méta-analyses à effets aléatoires ont été réalisées séparément pour les interventions ciblant des symptômes spécifiques et pour des interventions universelles plus générales. Cette revue documentaire a été enregistrée sur PROSPERO (CRD42023434878).

**Résultats:**

La méta-analyse comprenait 16 essais d’interventions ciblées (*n* = 2356) et 11 essais d’interventions universelles (*n* = 3378) qui satisfaisaient aux critères d’inclusion. Les enfants et les adolescents ayant bénéficié d’interventions ciblées ont fait état de beaucoup moins de symptômes de stress post-traumatique après l’intervention que les membres du groupe témoin. Ces effets positifs se sont maintenus lors du suivi. Des bénéfices ont également été observés concernant les symptômes dépressifs. En outre, les interventions universelles étaient associées à une réduction importante des symptômes de stress post-traumatique. Des analyses de modérateurs ont mis en évidence que les résultats n’étaient pas fortement influencés par le niveau de revenu du pays, par le recours à des thérapeutes professionnels ou non ou par le fait que le traumatisme ait été causé par des catastrophes naturelles ou d’origine humaine. Cependant, une hétérogénéité considérable et un risque élevé de biais ont été constatés d’une étude à l’autre.

**Conclusion:**

Les interventions de santé mentale et de soutien psychosocial, qu’elles soient ciblées ou universelles, étaient associées à une diminution des symptômes de stress post-traumatique chez les enfants et les adolescents se trouvant dans des situations de crise humanitaire, par rapport aux témoins passifs. Les interventions menées par des non-spécialistes ayant également donné de bons résultats, le partage des tâches pourrait être envisagé dans un contexte de ressources limitées.

## Resumen

**Objetivo:**

Evaluar la eficacia de las intervenciones de apoyo psicosocial y de salud mental para abordar los síntomas de estrés postraumático en niños, niñas y adolescentes en contextos humanitarios, mediante análisis separados de intervenciones específicas y universales.

**Métodos:**

Se realizaron búsquedas sistemáticas en las bases de datos MEDLINE, Web of Science, PTSDpubs y PsycInfo de ensayos aleatorizados controlados en contextos humanitarios que incluyeran a personas de 18 años o menos y compararan intervenciones psicológicas con controles pasivos. Se llevaron a cabo metanálisis de efectos aleatorios por separado para intervenciones dirigidas a síntomas específicos y para intervenciones universales más generales. La revisión fue registrada en PROSPERO (CRD42023434878).

**Resultados:**

El metanálisis incluyó 16 ensayos de intervenciones específicas (*n* = 2356) y 11 de intervenciones universales (*n* = 3378) que cumplían los criterios de inclusión. Los menores que recibieron intervenciones específicas presentaron significativamente menos síntomas de estrés postraumático tras la intervención en comparación con los controles. Estos efectos positivos se mantuvieron en el seguimiento. También se observaron beneficios en los síntomas depresivos. Además, las intervenciones universales se asociaron con una reducción significativa de los síntomas de estrés postraumático. Los análisis de moderadores mostraron que los resultados no se vieron influidos significativamente por el nivel de ingresos del país, el uso de terapeutas legos o profesionales, ni por la causa del trauma (conflictos o desastres naturales). No obstante, se observó una considerable heterogeneidad y un alto riesgo de sesgo entre los estudios.

**Conclusión:**

Tanto las intervenciones específicas como las universales de apoyo psicosocial y salud mental se asociaron con una reducción de los síntomas de estrés postraumático en menores en contextos humanitarios, en comparación con los controles pasivos. Dado que las intervenciones administradas por personal no especializado también demostraron ser eficaces, se recomienda considerar la redistribución de tareas en entornos con recursos limitados.

## ملخص

الغرض تقييم فعالية تدخلات الصحة النفسية والدعم النفسي والاجتماعي في معالجة أعراض إجهاد ما بعد الصدمة لدى الأطفال والمراهقين في البيئات الإنسانية، مع تحليلات منفصلة للتدخلات الموجهة والشاملة.

الطريقة لقد قمنا بالبحث بشكل منهجي في قواعد بيانات MEDLINE، وWeb of Science، وPTSDpubs، وPsycInfo، عن التجارب العشوائية ذات الصلة والخاضعة للضوابط، والتي شملت أفرادًا تبلغ أعمارهم 18 عامًا أو أقل في البيئات الإنسانية، وقارنا التدخلات النفسية مع الضوابط السلبية. تم إجراء تحليلات تلوية ذات تأثير عشوائي بشكل منفصل، للتدخلات التي تستهدف أعراضًا محددة، وللتدخلات الشاملة الأكثر عمومية. تم تسجيل المراجعة في PROSPERO (CRD42023434878).

النتائج شمل التحليل التلوي 16 تجربة للتدخلات المستهدفة (ن = 2356)، و11 تجربة للتدخلات الشاملة (ن = 3378)، التي استوفت معايير الإدراج. إن الأطفال والمراهقين الذين تلقوا تدخلات مستهدفة، أبلغوا عن أعراض أقل بشكل ملموس من إجهاد ما بعد الصدمة، بعد التدخل مقارنةً بالضوابط. واستمرت هذه الآثار الإيجابية عند المتابعة. كما لوحظت فوائد لأعراض الاكتئاب. بالإضافة إلى ذلك، ارتبطت التدخلات الشاملة بأعراض أقل بشكل ملموس من إجهاد ما بعد الصدمة. أظهرت تحليلات الوسيط أن النتائج لم تتأثر بشكل ملموس بمستوى دخل الدولة، أو استخدام المعالجين غير المتخصصين أو المحترفين، أو ما إذا كانت الصدمة ناجمة عن كوارث من صنع الإنسان أو طبيعية. ومع ذلك، لوحظ عدم تجانس كبير وارتفاع خطر التحيز عبر الدراسات.

الاستنتاج ارتبطت كل من تدخلات الصحة العقلية والدعم النفسي والاجتماعي المستهدفة والشاملة بأعراض أقل من إجهاد ما بعد الصدمة بين الأطفال والمراهقين في البيئات الإنسانية مقارنةً بالضوابط السلبية. ونظرًا لأن التدخلات التي قدمها غير المتخصصين كانت ناجحة أيضًا، فقد يكون من الممكن النظر في مشاركة المهام حيث تكون الموارد نادرة.

## 摘要

**目的:**

通过对针对性和普适性干预措施分别开展分析，评估为治疗人道主义环境下儿童和青少年的创伤后应激症状而采取的心理健康和社会心理支持干预措施的有效性。

**方法:**

我们系统地搜索了医学文献分析和检索系统在线 (MEDLINE)、Web of Science、PTSDpubs 和 PsycInfo 数据库，以查找针对人道主义环境下 18 岁或以下受影响人员开展的相关随机对照试验，并对比了含被动对照组的心理干预研究。对基于特定症状的针对性干预措施和更具普适性的干预措施分别进行了随机效应荟萃分析。我们还在国际前瞻性系统综述注册平台 (PROSPERO) 上注册了该系统评价（注册号：CRD42023434878）。

**结果:**

我们将符合纳入标准的 16 项针对性干预措施相关试验 (n = 2356) 和 11 项普适性干预措施相关试验 (n = 3378) 纳入了荟萃分析之中。根据报告结果，与被动对照组相比，接受了针对性干预措施治疗的儿童和青少年经干预后的创伤后应激症状明显有所减少。在随访时也还能维持这些积极效应。同时，我们发现抑郁症状也有所改善。此外，普适性干预措施也可明显减少创伤后应激症状。调节系数分析结果显示，国家收入水平、由非专业还是专业治疗师提供服务或者创伤是因人为因素还是自然灾害所致都对干预结果影响不大。但是，我们发现所有这些研究之间存在非常明显的异质性和较高的偏倚风险。

**结论:**

与被动对照组相比，针对性和普适性心理健康和社会心理支持干预措施都有助于减少人道主义环境下儿童和青少年的创伤后应激症状。由于非专业治疗师提供的干预措施同样成功见效，在资源匮乏的地区可考虑分摊干预任务。

## Резюме

**Цель:**

Оценить эффективность психиатрических вмешательств и психологической поддержки в вопросах облегчения симптомов посттравматического стресса у детей и подростков, находящихся в условиях гуманитарного кризиса, с раздельным анализом для вмешательств целенаправленного и общего характера.

**Методы:**

Авторы провели систематический поиск в базах MEDLINE, Web of Science, PTSDpubs и PsycInfo для выявления релевантных рандомизированных клинических контролируемых исследований, которые удовлетворяли следующим критериям: включали лиц в возрасте до 18 лет, проводились в условиях гуманитарного кризиса и сравнивали психологические методы лечения с пассивным контролем. Метаанализ по модели случайных эффектов был выполнен по отдельности для вмешательств, осуществлявшихся целенаправленно в отношении конкретных симптомов, и для вмешательств более общего характера. Обзор зарегистрирован на PROSPERO (CRD42023434878).

**Результаты:**

В метаанализ было включено 16 исследований целенаправленных вмешательств (n = 2536) и 11 исследований вмешательств общего характера (n = 3378), которые соответствовали критериям включения. Дети и подростки, которым проводились целенаправленные вмешательства, демонстрировали значительно меньшие проявления симптомов посттравматического стресса по сравнению с контрольной группой. Положительный эффект сохранялся в процессе последующего наблюдения. Отмечалась также польза в отношении депрессивных симптомов. Кроме того, вмешательства общего характера ассоциировались со значительно меньшими проявлениями симптомов посттравматического стресса. Модераторные анализы показали, что на результаты не оказывали существенного влияния такие факторы, как уровень дохода страны, использование для вмешательств услуг профессиональных терапевтов или обычных людей, а также характер причины травмы (антропогенные конфликты или природные бедствия). Однако для исследований были отмечены значительная разнородность и высокий риск предвзятости.

**Вывод:**

Как целенаправленные, так и общие психиатрические вмешательства и психологическая поддержка ассоциировались с меньшими проявлениями симптомов посттравматического стресса у детей и подростков, находящихся в условиях гуманитарного кризиса, по сравнению с пассивным контролем. Вмешательства, проводимые неспециалистами, были также успешными, поэтому в условиях нехватки соответствующих ресурсов следует рассматривать возможность совместного решения подобных задач.

## Inroduction

Humanitarian crises can threaten the health, safety, security and wellbeing of communities.^1^ In 2024, over 460 million children globally were affected by conflict and, in 2021, an estimated one billion children were at risk from the effects of climate change, including hurricanes, flooding, famine and drought.^2^^,^^3^ Population displacement restricts people’s access to essential services, shelter and health care, and conflict threatens their security.^4^ Children are particularly vulnerable due to the impact on their development,^5^^,^^6^ and they have a higher risk of mental disorders, including depression and anxiety.^7^^,^^8^ Post-traumatic stress disorder (PTSD) is common and is characterized by prolonged psychological distress, flashbacks, nightmares, hyperarousal, avoidance of trauma-related stimuli and changes in mood or cognition.^7^^,^^9^ Research shows that childhood trauma affects mental health in adulthood and that early support may be beneficial,^10^^,^^11^ which highlights the need for timely interventions.

Given their increased risk of post-traumatic stress symptoms,^12^^–^^14^ children in humanitarian settings may benefit from interventions such as mental health and psychosocial support, which has been defined as, “any type of local or outside support that aims to protect or promote psychosocial well-being and/or prevent or treat mental disorder.”^15^ Interventions can be targeted, with a focus on clinically significant symptoms, or universal and offered irrespective of the symptom burden. Specialist mental health services may be limited in low-resource settings. However, access to mental health and psychosocial support is broadened when it is provided by non-specialists or community workers or in a group setting.^15^ For children, interventions are often delivered to groups in, for example, schools.^16^

Humanitarian organizations and policy-makers need reliable evidence to inform resource allocation and to identify the most effective, contextually appropriate and sustainable interventions.^17^ The aim of our review was to investigate the effectiveness of mental health and psychosocial support interventions in addressing post-traumatic stress symptoms among children and adolescents in humanitarian settings. As depression and anxiety frequently co-occur in children with PTSD,^9^ we also examined whether interventions targeting PTSD can have a beneficial effect on these comorbid symptoms.

By focusing on studies that employed inactive or passive controls, such as waitlist controls or children not exposed to interventions, we were able to evaluate the overall efficacy of mental health and psychosocial support interventions while considering the spontaneous remission of post-traumatic stress symptoms.^18^ In addition, we confined our review to randomized controlled trials to ensure we included the highest level of evidence. Our priority was in situ studies because we aimed to evaluate the efficacy of mental health and psychosocial support in insecure and often resource-poor humanitarian settings.

## Methods

We systematically searched MEDLINE, Web of Science, PTSDpubs (ProQuest, Ann Arbor, United States of America) and PsycInfo databases from inception to 21 March 2025, without language restrictions, for articles on psychological interventions for treating post-traumatic stress symptoms in children in humanitarian settings. Searches were limited to titles and abstracts. The searches used the following criteria: (i) the study type was the randomized controlled trial; (ii) participants were children or adolescents; (iii) symptoms were post-traumatic stress symptoms; (iv) the setting was a humanitarian crisis, such as a conflict, population displacement or natural disaster; and (v) interventions were psychological or psychosocial (Box 1). Studies included in systematic reviews identified were screened for eligibility.^19^^–^^28^

Box 1 **Search strategy, meta-analysis of psychological interventions for post-traumatic stress in children and adolescents in humanitarian crises, 2002–2024**

The Medline, Web of Science, PTSDpubs and PsycINFO databases were searched for articles up until the date of the search on 26 June 2023. The databases were searched again on 10 February 2024 and on 21 March 2025, with search terms updated to include “student”, “boy*”, “girl*”, and “treatment”. Only titles and abstracts were searched and each search was adapted to the specific database’s indexing language. In general, the search terms were:

study type (randomized OR randomized OR controlled OR control);

AND

study participants (child* OR adolesc* OR minor OR youth OR student OR boy* OR girl*);

AND

symptom type (ptsd OR post traumatic stress OR posttraumatic stress OR post-traumatic stress);

AND

study setting (global OR humanitarian OR immig* OR refugee* OR war OR displac* OR conflict OR famine OR drought OR earthquake OR tsunami OR mass trauma OR disaster OR migra* OR evacu* OR asylum OR stateless* OR emergen* OR violence OR abuse* OR torture OR catastroph* OR flood OR hurricane OR cyclone* OR landslide* OR land slide* OR mass casualt* OR tidal wave* OR volcan* OR resettle*);

AND

intervention (therapy OR intervention OR psyc* OR counselling OR counseling OR psychotherapy OR treatment).

Notes: The asterisk indicates a wildcard search to find variations on the search term. No language restrictions were applied. Studies included in systematic reviews identified were screened for eligibility.

Studies were included in the meta-analysis if they: (i) were conducted in a humanitarian setting, including settings with internally displaced people; (ii) involved the random allocation of participants; (iii) had at least one study arm that included a psychological or psychosocial intervention (e.g. cognitive-behavioural therapy or group therapy) whose primary aim was to treat post-traumatic stress symptoms; (iv) had an inactive or passive control group; (v) had a sample mean age under 19 years; and (vi) had outcome data for 10 or more participants per group or arm. Studies were excluded if they: (i) were conducted in a resettlement country or an immigration centre; (ii) compared interventions without a control group; or (iii) did not report trauma-specific outcome measures.

Study outcomes were single post-intervention measures of post-traumatic stress symptoms made at any time and subsequent follow-up measures, if evaluated, and post-intervention and follow-up measures of depression and anxiety, where reported. 

### Data extraction

After removing duplicate publications, two authors screened the titles, abstracts and full texts of articles. Disagreement was resolved through discussion with a third author. Where more than one intervention was compared with the control group, the intervention based on cognitive-behavioural therapy was used for the comparison to avoid data duplication. Two authors independently extracted quantitative and descriptive data.

We explored the following moderator variables: (i) the cause of the trauma (i.e. human-made versus natural disaster); (ii) the individuals delivering the intervention (i.e. professional versus lay therapists); (iii) the participants’ age; (iv) number of intervention sessions; (v) country income; and (vi) the risk of study bias.

### Data analysis

We performed a random effects meta-analysis using the metafor package in R, version 4.4.1 (The R Foundation, Vienna, Austria).^29^ Between-group effect sizes were calculated using Hedges’ *g*. Where available, means and standard deviations were used. Otherwise, Cohen’s *d* was extracted or was derived from other reported statistics. To explore the level of heterogeneity, we derived Cochran’s *Q* statistic, the *I^2^* statistic and prediction intervals. Forest and funnel plots are also presented. Details are available from the online repository.^30^

The risk of bias was assessed independently by two authors using the Cochrane risk-of-bias tool, version 2,^31^ and by examining randomization, deviations from the intended intervention, missing data, how outcomes were measured, and reporting bias. A study with concerns about bias in any domain was classified as either having some concerns or being high risk, depending on the nature of the flaws. A study rated high risk in any domain was deemed high risk overall. A table detailing the risk of bias assessments is available in the online repository.

The possibility of publication bias affecting the primary study outcome was assessed by testing for asymmetry (i.e. Egger’s test)^32^ and visual inspection of funnel plots. If required, a trim-and-fill procedure was used to correct for possible publication bias.^33^

The meta-analysis was registered in the PROSPERO registry (CRD42023434878) and followed PRISMA reporting guidelines.^34^

## Results

**Fig. 1:**
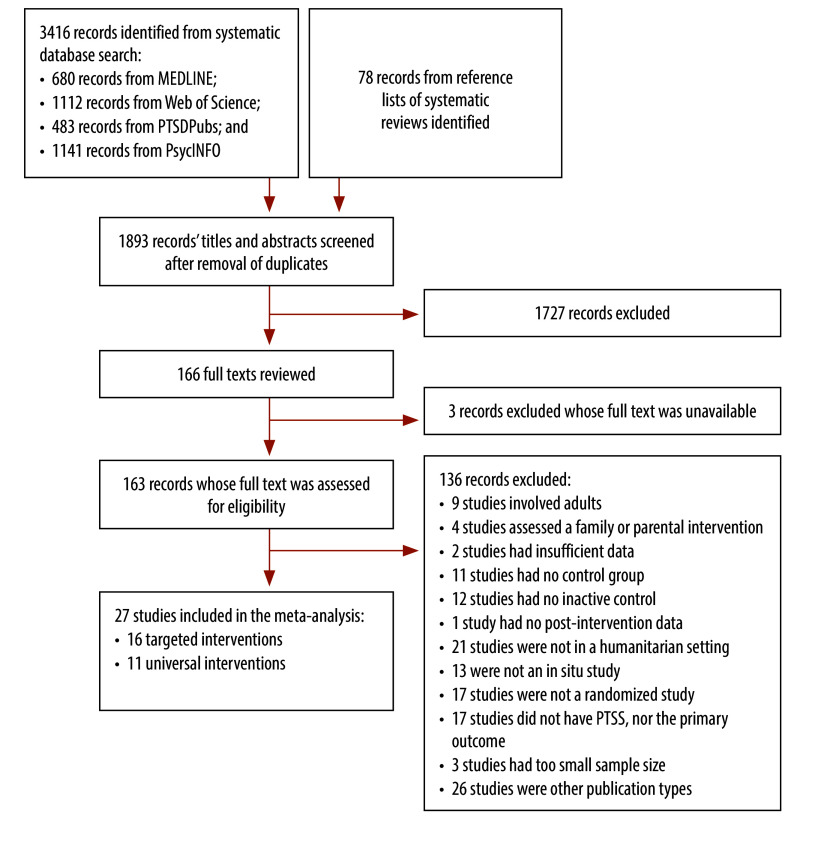
Study selection flowchart, meta-analysis of psychological interventions for post-traumatic stress in children and adolescents in humanitarian crises, 2002–2024

Following the screening of 1893 titles and abstracts, 166 publications were identified for full text review, 163 of which were successfully retrieved (Fig. 1). Of the 163, 27 were eligible for inclusion in the meta-analysis.^37^^–^^63^ Details of the context and participants of the 27 studies are presented in Table 1 and details of interventions, outcome measures and the risk of bias are shown in Table 2. Table 3 and Table 4 report the results of the meta-analyses of targeted and universal interventions, respectively.

PTSS: post-traumatic stress symptoms.

Notes: the two studies excluded for insufficient data lacked the requisite statistical data and attempts to derive Cohen’s *d* produced results that could not be reliably interpreted.^35^^,^^36^ The study authors did not respond to requests for clarification. Other publication types were: moderator analysis (4); cross-sectional study (1); correction (3); analysis of secondary data (6); case–control study (1); case series (1); observational study (1); quasi-experimental study (1); study protocol (1); commentary (4); book (1); and qualitative study (2).

**Table 1:** Study context and participants, meta-analysis of psychological interventions for post-traumatic stress in children and adolescents in humanitarian crises, 2002–2024

Study authors and year	Context				Participants				
	Territory	Territory income status during study^a^	Setting for intervention		Cause of trauma	Sample size	Mean age (range), years	Female, %	Proportion meeting criteria for PTSD, %
**Targeted interventions **									
Chemtob et al., 2002^42^	Hawaii, United States	High	School		Hurricane	32	8.4 (6 to 12)	68.7	ND
Gordon et al., 2008^45^	Kosovo	Lower middle	School		Conflict	82	16.3 (14 to 18)	75.7	ND
Sadeh et al., 2008^48^	Israel	High	School		Conflict	292	4.5 (2 to 7)	45.2	ND
Tol et al., 2008^50^	Indonesia	Lower middle	School		Conflict	403	9.9 (7 to 15)	48.6	ND
Lesmana et al., 2009^52^	Indonesia	Lower middle	Not stated		Terrorism	226	9.8 (6 to 12)	52.7	ND
Jordans et al., 2010^46^	Nepal	Low	School		Conflict	325	12.7 (11 to 14)	48.6	ND
Ertl et al., 2011^51^	Uganda	Low	Internally displaced persons camp		Conflict	57	18.4 (12 to 25)	55.3	ND
Barron et al., 2013^41^	West Bank and Gaza Strip	Lower middle	School		Conflict	140	11.1 (11 to 14)	45	ND
Chen et al., 2014^43^	China	Upper middle	School		Earthquake	28	14.5 (ND)	68	ND
Tol et al., 2014^49^	Burundi	Low	School		Conflict	329	12.3 (8 to 17)	48	ND
Pityaratstian et al., 2015^47^	Thailand	Upper middle	School and beach		Tsunami	36	12.3 (10 to 15)	72.2	ND
Barron et al., 2016^40^	West Bank and Gaza Strip	Lower middle	School		Conflict	154	13.57 (11 to 15)	60	ND
Amin et al., 2020^39^	Pakistan	Lower middle	School		Flood	75	11.4 (7 to 13)	35	ND
Ahmadi et al., 2022^37^	Afghanistan	Low	School		Terrorism	80	16.1 (12 to 18)	100	ND
Getanda & Vostanis, 2022^44^	Kenya	Lower middle	School		Conflict	50	ND (14 to 17)	59	ND
Ahmadi et al., 2024^38^	Afghanistan	Low	School		Conflict	43	15.2 (ND)	99	ND
**Universal interventions**									
Berger et al., 2007^53^	Israel	High	School		Terrorism	142	ND (7 to 11)	45.8	7.7
Berger & Gelkopf, 2009^54^	Sri Lanka	Lower middle	School		Tsunami	166	ND (9 to 14)	47.6	32.5
Gelkopf & Berger, 2009^57^	Israel	High	School		Terrorism	107	13.1 (12 to 14.5)	0	0
Berger et al., 2012^55^	Israel	High	School		Conflict	154	12.8 (11 to 13)	53.9	43.5
Peltonen et al., 2012^60^	West Bank and Gaza Strip	Lower middle	School		Conflict	225	11.4 (10 to 14)	36	58
Qouta et al., 2012^61^	West Bank and Gaza Strip	Lower middle	School		Conflict	482	11.3 (10 to 13)	49.4	54
McMullen et al., 2013^58^	Democratic Republic of the Congo	Low	School		Conflict	50	15.8 (13 to 17)	0	ND
O’Callaghan et al., 2013^59^	Democratic Republic of the Congo	Low	School		Conflict	52	16.0 (12 to 17)	100	ND
O’Callaghan et al., 2014^63^	Democratic Republic of the Congo	Low	Village		Conflict	159	13.4 (7 to 18)	45	ND
Ruggiero et al., 2015^62^	United States	High	Online		Tornado	621	14.5 (12 to 17)	51	7.7
Dhital et al., 2019^56^	Nepal	Lower middle	School		Earthquake	1220	12.9 (ND)	53.5	ND

ND: not determined; PTSD: Post-traumatic stress disorder.

^a^ Each country’s income status was determined from the World Bank’ income classification for the year of study publication.^64^

**Table 2:** Study interventions, outcome measures and risk of bias, meta-analysis of psychological interventions for post-traumatic stress in children and adolescents in humanitarian crises, 2002–2024

Study authors and year	Intervention							Outcomes					Risk of bias^a^
	Type	Description	Therapist	Group or individual	No. sessions	Control treatment or group		Time to first measure	Follow-up period	PTSD symptom measure	Depression symptom measure	Anxiety symptom measure	
**Targeted interventions**													
Chemtob et al., 2002^42^	Eye-movement desensitization and reprocessing	Uses techniques to help the brain process memories and reduce their emotional impact through guided eye movements while recalling traumatic experiences	Professional^b^	Individual	3	Waiting list group		Not stated	6 months	Children’s Reaction Inventory (interview)	Children’s Depression Inventory (self-reported)	Revised Children's Manifest Anxiety Scale (self-reported)	High
Gordon et al., 2008^45^	Mind–body skills group programme	Combines several mind–body modalities together with self-expression through the spoken and written word and in drawings and movement	Teachers	Group	12	Waiting list group		Immediate	NA	Harvard Trauma Questionnaire (self-reported)	NA	NA	Some concerns
Sadeh et al., 2008^48^	Huggy puppy	Involves giving the child a toy dog to take care of, “to empower them and facilitate their active coping with stressful situations”	Psychologists^b^	Group	Ongoing	No visits		2 months	NA	Stress Reaction Checklist (parent report)	NA	NA	High
Tol et al., 2008^50^	Classroom-based intervention	See Jordans 2010^46^	Lay people	Group	15	Waiting list group		1 week	6 months	Child Post-Traumatic Stress Scale (self-reported)	Depression Self-Rating Scale (self-reported)	Screen for Child Anxiety Related Disorders, 5-item version (self-reported)	Some concerns
Lesmana et al., 2009^52^	Spiritual-hypnosis-assisted treatment	Uses hypnosis in a group setting, with sessions including guidance during a trance state	Study researcher^b^	Group	1	No treatment		2 years	NA	Developed by study researchers (interview)	NA	NA	High
Jordans et al., 2010^46^	Classroom-based intervention	Based on concepts from creative-expressive and experiential therapy, cooperative play and cognitive-behavioural therapy	Local research assistants	Group	15	Waiting list group		Not stated	NA	Child PTSD Symptom Scale (interview)	Depression Self-Rating Scale (self-reported)	Screen for Child Anxiety Related Disorders, 5-item version (self-reported)	Some concerns
Ertl et al., 2011^51^	Narrative exposure therapy	Aims to reduce PTSD symptoms and improve emotional functioning through creating a coherent life narrative and structured storytelling	Locally trained therapists	Group	8	Waiting list group		3 months	6 months, 12 months^c^	Clinician-Administered PTSD Scale (interview)	Mini International Neuropsychiatric Interview (interview)	NA	Some concerns
Barron et al., 2013^41^	Teaching recovery techniques	Offers strategies for managing stress and emotional difficulties while teaching coping skills and emotional regulation	Trained counsellors	Group	5	Waiting list group		2 weeks	NA	Child Revised Impact of Events Scale, 13-item version (self-reported)	Depression Self-Rating Scale (self-reported)	NA	High
Chen et al., 2014^43^	Short-term cognitive behavioural therapy	Addresses negative thought patterns and behaviours and teaches healthier thinking and coping strategies for stress, anxiety and depression	Not stated	Group	6	No intervention		Immediate	3 months	Child Revised Impact of Events Scale, 13-item version (self-reported)	Center for Epidemiologic Studies Depression Scale (interview)	NA	High
Tol et al., 2014^49^	Classroom-based intervention	See Jordans 2010^46^	Paraprofessionals	Group	15	Waiting list group		1 week	3 months	Child Post-Traumatic Stress Scale (self-reported)	Depression Self-Rating Scale (self-reported)	NA	Some concerns
Pityaratstian et al., 2015^47^	Teaching recovery techniques	See Barron 2013^41^	Psychiatrist^b^	Group	3	Waiting list group		Immediate	1 month	Child Revised Impact of Events Scale, Thai version (self-reported)	NA	NA	Some concerns
Barron et al., 2016^40^	Teaching recovery techniques	See Barron 2013^41^	Trained counsellors	Group	5	Waiting list group		2 weeks	NA	Child Revised Impact of Events Scale, 8-item version (self-reported)	Depression Self-Rating Scale (self-reported)	NA	Some concerns
Amin et al., 2020^39^	Support for students exposed to trauma	Instruction on stress management, emotional regulation and social support, focusing on improving mental well-being in a group setting	Clinically trained personnel	Group	10	No treatment		Immediate	NA	Child PTSD Symptom Scale self-report for the Diagnostic and Statistical Manual of Mental Disorders, Fifth Edition (self-reported)	NA	NA	High
Ahmadi et al., 2022^37^	Trauma-focused cognitive-behavioural therapy	Combination of cognitive-behavioural techniques with trauma-sensitive methods to process traumatic memories and reduce anxiety and depression	Community facilitator	Group	5	No contact		Not stated	3 months	Child Revised Impact of Events Scale, 13-item version (self-reported)	NA	NA	High
Getanda & Vostanis, 2022^44^	Writing for recovery	Writing exercises to help individuals process and heal from trauma: the intervention supports mental health and personal growth through self-reflection and creative expression	Paraprofessionals	Group	6	Waiting list group		Immediate	1 week	Child Revised Impact of Events Scale, 13-item version (self-reported)	Depression Self-Rating Scale (self-reported)	Revised Children's Manifest Anxiety Scale (self-reported)	High
Ahmadi et al., 2024^38^	Trauma-focused cognitive-behavioural therapy	See Ahmadi 2022^37^	Trained facilitator	Group	7	Waiting list group		Immediate	NA	Child Revised Impact of Events Scale, 13-item version (self-reported)	NA	NA	Some concerns
**Universal interventions**													
Berger et al., 2007^53^	Overshadowing the threat of terrorism	Uses psychoeducational and skills training with meditative practices, bio-energy exercises, art therapy and narrative techniques for reprocessing traumatic experiences	Teachers	Group	8	Waiting list group		2 months	NA	University of California at Los Angeles Posttraumatic Stress Disorder Reaction Index (self-reported)	NA	Screen for Child Anxiety Related Disorders (self-reported)	Some concerns
Berger & Gelkopf, 2009^54^	Enhancing resiliency among students experiencing stress	Teacher-led intervention that provides educational resources and strategies for emotion regulation and stress management	Teachers	Group	12	Waiting list group		3 months	NA	University of California at Los Angeles Posttraumatic Stress Disorder Reaction Index (self-reported)	Beck Depression Inventory (self-reported)	NA	Some concerns
Gelkopf & Berger, 2009^57^	Enhancing resiliency among students experiencing stress	See Berger and Gelkopf 2009^54^	Teachers	Group	12	Waiting list group		3 months	NA	University of California at Los Angeles Posttraumatic Stress Disorder Reaction Index (self-reported)	Beck Depression Inventory (self-reported)	NA	Some concerns
Berger et al., 2012^55^	Enhancing resiliency among students experiencing stress	See Berger and Gelkopf 2009^54^	Teachers	Group	16	Waiting list group		1 month	NA	University of California at Los Angeles Posttraumatic Stress Disorder Reaction Index (self-reported)	NA	Screen for Child Anxiety Related Disorders (self-reported)	Some concerns
Peltonen et al., 2012^60^	School mediation intervention	Aims to improve social functioning through problem-solving, conflict resolution and dialogue skills, while enhancing mental health by encouraging peer support and reducing disruptive or aggressive behaviour	Teachers, field supervisors and students	Group	Ongoing	School as usual		8 months	NA	Child Post-Traumatic Stress Reaction Index (self-reported)	Children’s Depression Inventory (self-reported)	NA	Some concerns
Qouta et al., 2012^61^	Teaching recovery techniques	See Barron 2013^41^	Psychologists^b^	Group	8	Waiting list group		Not stated	6 months	Child Revised Impact of Events Scale, 13-item version (self-reported)	Depression Self-Rating Scale (self-reported)	NA	Some concerns
McMullen et al., 2013^58^	Trauma-focused cognitive-behavioural therapy	See Ahmadi 2022^37^	Study authors and counsellors^b^	Group	15	Waiting list group		7 weeks	NA	University of California at Los Angeles Posttraumatic Stress Disorder Reaction Index (self-reported)	NA	NA	Some concerns
O’Callaghan et al., 2013^59^	Trauma-focused cognitive-behavioural therapy	See Ahmadi 2022^37^	Social workers^b^	Group and individual	15	Waiting list group		7 weeks	NA	University of California at Los Angeles Posttraumatic Stress Disorder Reaction Index (self-reported)	NA	NA	Some concerns
O’Callaghan et al., 2014^63^	Intervention developed by study authors	Intervention manual based on a youth life-skills leadership programme, videos to address stigma, and features of trauma-focused cognitive-behavioural therapy	Lay facilitators	Group	8	Waiting list group		4 weeks	NA	Child Revised Impact of Events Scale, 8-item version (self-reported)	NA	NA	Some concerns
Ruggiero et al., 2015^62^	Bounce Back Now	An online intervention in which individuals selected from four online modules that addressed PTSD, smoking, alcohol use and depression, and that used behavioural strategies and multimedia content	Online portal	Individual	Up to four modules	Assessment only		4 months	12 months	Negative Symptom Assessment for PTSD (interview)	Negative Symptom Assessment for depression (interview)	NA	Some concerns
Dhital et al., 2019^56^	Psychosocial support training for teachers	Uses a training manual developed for teachers enabling them to provide students with psychosocial support	Teachers	Group	Ongoing	Use of untrained teachers		6 months	NA	Child PTSD Symptom Scale (self-reported)	Depression Self-Rating Scale (self-reported)	NA	High

NA: not applicable; PTSD: post-traumatic stress disorder.

^a^ Risk of bias was assessed using the Cochrane risk-of-bias tool version 2.^31^

^b^ Intervention delivered by specialists.

^c^ Only follow-up measures at 12 months were included in the meta-analysis.

**Table 3:** Effect of targeted psychological interventions for post-traumatic stress in children and adolescents in humanitarian crises, 2002–2024

Variable	No. studies	No. participants^a^		Pooled effect of intervention on symptoms^b^			Study heterogeneity		Significance of moderator,^c^ *P*-value
				Hedges’ *g* (95% CI)	95% prediction interval^d^		Cochran’s *Q* statistic	*I^2^*, %	
**Post-traumatic stress symptoms (post-intervention data)**	16	2277		−0.78 (−1.08 to −0.48)	−1.91 to 0.34		118.5^e^	90	NA
**Post-traumatic stress symptoms (follow-up data)**	8	885		−0.91 (−1.46 to −0.35)	−2.46 to 0.64		49.8^f^	92	NA
**Depression (post-intervention data)**	9	1452		−0.37 (−0.69 to −0.04)	−1.28 to 0.54		46.4^g^	87	NA
**Depression (follow-up data)**	6	803		−0.49 (−0.92 to −0.05)	−1.51 to 0.53		18.3^g^	85	NA
**Anxiety (post-intervention data)**	4	798		−0.24 (−0.82 to 0.34)	−1.45 to 0.98		16.9	92	NA
**Anxiety (follow-up data)**	3	448		−0.14 (−0.32 to 0.05)	−0.32 to 0.05		0.90	0	NA
**Moderator**									
Cause of trauma									0.89
Human-made event	12	2112		−0.76 (−1.04 to −0.48)	−1.68 to 0.16		81.0^e^	88	
Natural disaster	4	165		−0.80 (−1.80 to 0.20)	−2.93 to 1.33		30.5	88	
Country income status^h^									0.58
Low or lower middle	7	911		−0.91 (−1.56 to −0.27)	−2.67 to 0.85		83.7^f^	95	
Upper middle or high	9	1366		−0.75 (−0.96 to −0.54)	−1.25 to −0.25		18.8^e^	62	
Therapist type									0.61
Professional	4	586		−0.68 (−1.20 to −0.16)	−1.73 to 0.38		14.7^g^	83	
Lay person	11	1669		−0.85 (−1.25 to −0.46)	−2.15 to 0.44		97.0^e^	93	
Risk of bias^i^									0.026
High	8	902		−1.09 (−1.53 to −0.64)	−2.30 to 0.13		38.4^e^	87	
Some concerns	8	1375		−0.49 (−0.79 to −0.19)	−1.28 to 0.30		40.3^f^	84	
Mean age of participants, years									0.48
≥ 13	7	476		−0.91 (−1.27 to −0.55)	−1.76 to −0.05		17.5^e^	69	
< 13	9	1801		−0.69 (−1.14 to −0.25)	−2.04 to 0.65		93.3	95	
No. intervention sessions^j^	NA	NA		NA	NA		NA	NA	0.46

CI: confidence interval; NA: not applicable.

^a^ The number of participants was based on data reported by individual studies; the analysis did not include participants who dropped out or whose results were not reported.

^b^ The intervention groups were compared with inactive or passive control groups.

^c^ The significance of the moderators was estimated using an omnibus test.^29^

^d^ The prediction interval is the range within which the effect of a single, new intervention on symptoms is likely to fall.

^e^ *P* < 0.001.

^f^ *P* < 0.01.

^g^ *P* < 0.05.

^h^ Country income status was determined from the World Bank’ income classification for the year of study publication.^64^

^i^ Risk of bias was assessed using the Cochrane risk-of-bias tool version 2.^31^

^j^ As the number of intervention sessions was treated as a continuous variable for the moderator analysis, no subgroup data are available.

Note: the moderator analysis is of post-intervention PTSS outcomes.

**Table 4:** Effect of universal psychological interventions for post-traumatic stress in children and adolescents in humanitarian crises, 2002–2024

Variable	No. studies	No. participants^a^		Pooled effect of intervention on symptoms^b^			Study heterogeneity		Significance of moderator,^c^ *P*-value
				Hedges’ *g* (95% CI)	95% prediction interval^d^		Cochran’s *Q* statistic	*I^2^, %*	
**Post-traumatic stress symptoms (post-intervention data)**	11	3010		−0.75 (−1.23 to −0.26)	−2.37 to 0.88		148.6^e^	97	NA
**Post-traumatic stress symptoms (follow-up data)**	2	726		−0.17 (−0.32 to −0.03)	−0.32 to −0.03		0.05^f^	0	NA
**Depression (post-intervention data)**	6	2455		−0.13 (−0.31 to 0.06)	−0.55 to 0.29		16.9	76	NA
**Depression (follow-up data)**	2	726		−0.04 (−0.33 to 0.25)	−0.49 to 0.41		3.8	74	NA
**Anxiety (post-intervention data)**	2	296		−0.52 (−1.37 to 0.33)	−1.95 to 0.91		12.1	92	NA
**Moderator**									
Cause of trauma									0.42
Human-made event	8	1369		−0.88 (−1.49 to −0.27)	−2.66 to 0.90		86.1^e^	96	
Natural disaster	3	1641		−0.42 (−1.23 to 0.38)	−2.02 to 1.17		48.5	98	
Country income status^g^									0.55
Low or lower middle	7	2202		−0.89 (−1.65 to −0.13)	−2.99 to 1.21		115.0^f^	98	
Upper middle or high	4	808		−0.54 (−0.99 to −0.08)	−1.50 to 0.42		32.1^f^	88	
Therapist type									0.055
Professional	3	582		−1.56 (−3.08 to −0.04)	−4.55 to 1.43		63.1^f^	96	
Lay person	8	2428		−0.48 (−0.82 to −0.14)	−1.44 to 0.49		83.5^e^	93	
Mean age of participants, years									0.159
≥ 13	5	771		−1.10 (−2.08 to −0.12)	−3.45 to 1.24		73.6^f^	97	
< 13	5	2073		−0.33 (−0.70 to 0.04)	−1.19 to 0.53		32.4	93	
No. intervention sessions^h^	NA	NA		NA	NA		NA	NA	< 0.005

CI: confidence interval; NA: not applicable.

^a^ The number of participants was based on data reported by individual studies; the analysis did not include participants who dropped out or whose results were not reported.

^b^ The intervention groups were compared with inactive or passive control groups.

^c^ The significance of the moderators was estimated using an omnibus test.^29^

^d^ The prediction interval is the range within which the effect of a single, new intervention on symptoms is likely to fall.

^e^ *P* < 0.01.

^f^ *P* < 0.05.

^g^ Country income status was determined from the World Bank’ income classification for the year of study publication.^64^

^h^ As the number of intervention sessions was treated as a continuous variable for the moderator analysis, no subgroup data are available.

Note: the moderator analysis is of post-intervention PTSS outcomes.

### Targeted interventions

The search identified 16 randomized controlled trials of targeted interventions,^37^^–^^52^ involving a total of 2356 children, that were published between 2002 and 2024 (Table 1). Overall, 53% (1260/2356) of participants were female. Across studies reporting full age data,^37^^–^^43^^,^^45^^–^^52^ the pooled mean age was 11.1 years (range of study means: 4.5 to 18.4 years). Fourteen studies were conducted in schools;^37^^–^^50^ 12 took place in the context of conflict or terrorism^37^^,^^38^^,^^40^^,^^41^^,^^44^^–^^46^^,^^48^^–^^52^ and four followed a natural disaster, including a flood, hurricane, earthquake and tsunami.^39^^,^^42^^,^^43^^,^^47^ The income classification of the study countries was low for five,^37^^,^^38^^,^^46^^,^^49^^,^^51^ lower-middle for seven,^39^^–^^41^^,^^44^^,^^45^^,^^50^^,^^52^ upper-middle for two,^43^^,^^47^ and high for two.^42^^,^^48^

Fifteen studies involved group interventions,^37^^–^^41^^,^^43^^–^^52^ with four delivered by specialists (Table 2).^42^^,^^47^^,^^48^^,^^52^ The number of intervention sessions ranged from one to 15, with one study involving an ongoing intervention.^48^ The most frequently used interventions were based on cognitive-behavioural therapy,^37^^,^^38^^,^^40^^,^^41^^,^^43^^,^^47^ or took place in classrooms.^37^^–^^50^

Post-traumatic stress symptoms were evaluated using a self-report scale in 11 studies,^37^^–^^41^^,^^43^^–^^45^^,^^47^^,^^49^^,^^50^ a diagnostic interview in four,^42^^,^^46^^,^^51^^,^^52^ and a parental report in one.^48^ Depressive symptoms were assessed using a self-report scale in seven studies,^40^^–^^42^^,^^44^^,^^46^^,^^49^^,^^50^ and a diagnostic interview in two.^43^^,^^51^ In all four cases, anxiety was evaluated using a self-report scale.^42^^,^^44^^,^^46^^,^^50^ Eight studies reported the follow-up of post-traumatic stress symptoms,^37^^,^^42^^–^^44^^,^^47^^,^^49^^–^^51^ at between 1 week and 12 months. Depressive symptoms were reported post-intervention in nine studies,^40^^–^^44^^,^^46^^,^^49^^–^^51^ and at follow-up in six.^42^^–^^44^^,^^49^^–^^51^ Anxiety symptoms were assessed post-intervention in four studies,^42^^,^^44^^,^^46^^,^^50^ and at follow-up in three.^42^^,^^44^^,^^50^

**Fig. 2:**
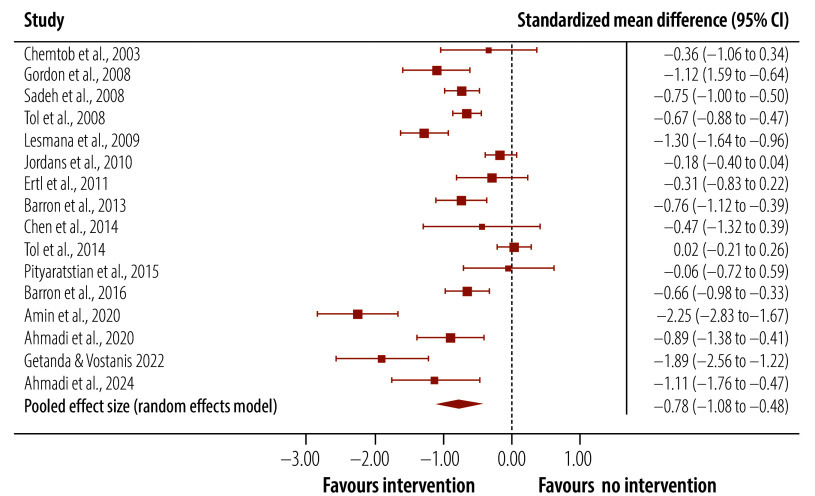
Effect of targeted interventions on post-traumatic stress symptoms in children and adolescents in humanitarian crises, post-intervention assessments, 2002–2024

**Fig. 3:**
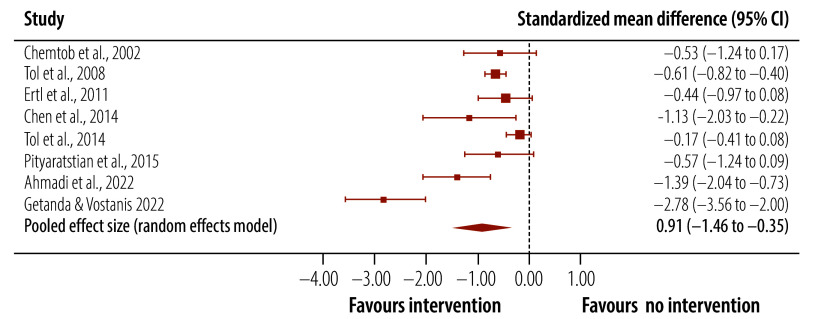
Effect of targeted interventions on post-traumatic stress symptoms in children and adolescents in humanitarian crises, follow-up assessments, 2002–2024

A meta-analysis of trials of targeted interventions that assessed post-traumatic stress symptoms post-intervention demonstrated better outcomes in the intervention than the control groups (Table 3): the pooled effect size was medium and Hedges’ *g* was −0.78 (95% confidence interval, CI: −1.08 to −0.48; Fig. 2). This benefit was also seen at follow-up (Hedges’ *g*: −0.91; 95% CI: −1.46 to −0.35; Fig. 3). The heterogeneity between studies was significant. However, an inspection of funnel plots and a statistical analysis did not indicate publication bias. For depression scores, there was a small effect size post-intervention (Hedges’ *g*: −0.37; 95% CI: −0.69 to −0.04), which was maintained at follow-up (Hedges’ *g*: −0.49; 95% CI: −0.92 to −0.05). No significant difference in anxiety symptoms between intervention and control groups was found.

CI: confidence interval.

Note: the pooled effect size is expressed as Hedges’ *g* (95% CI).

CI: confidence interval;

Note: the pooled effect size is expressed as Hedges’ *g* (95% CI).

There were some concerns about, or a high risk of, bias in all studies (Table 2). In particular, measurement of outcome was of some concern in all studies due to a lack of blinding. No study provided a prespecified plan to analyse outcomes. One study was regarded as having a high risk of bias during randomization because, although study counsellors were randomly allocated to deliver either the intervention or control conditions, they were responsible for selecting which classes to teach.^41^ One study was deemed to have a high risk of deviation from the study intervention because six of 16 participants in the intervention arm failed to complete the study.^43^ The effect size was significantly larger in studies with a high risk of bias than in those with some concern of bias (*P*-value: 0.026; Table 3).

A moderator analysis of the effect size of the intervention on post-traumatic stress symptoms post-intervention found no significant difference between human-made events and natural disasters (Table 3). However, the effect size did not reach significance in natural disasters, which may reflect the smaller sample size. No significant difference in effect size was observed for the number of intervention sessions, country income group, whether individuals delivering the intervention were specialists or not, or whether mean age was above or below 13 years. 

### Universal interventions

Eleven randomized controlled trials of universal interventions, involving a total of 3378 children, were identified (Table 1).^53^^–^^63^ They were published between 2007 and 2019. Overall, 49% (1639/3378) of participants were female. Across studies reporting full age data,^55^^–^^63^ the pooled mean age was 13.0 years (range of study means: 11.3 to 16.0 years); though, of note, two of the other studies reported ranges of 7 to 11 years and 9 to 14 years, respectively.^53^^,^^54^ Nine studies were conducted in schools.^53^^–^^61^ Three studies took place after a natural disaster (i.e. a tsunami, earthquake and tornado, respectively),^54^^,^^56^^,^^62^ and eight were conducted in the context of a conflict or terrorist attack. The income classification of the study countries was low for three,^58^^,^^59^^,^^63^ lower-middle for four,^54^^,^^56^^,^^60^^,^^61^ and high for four.^53^^,^^55^^,^^57^^,^^62^ Where reported, the frequency of post-traumatic stress symptoms in the study cohorts ranged from 0% to 58%.^53^^–^^55^^,^^57^^,^^60^^–^^62^

Nine studies involved group interventions,^53^^–^^58^^,^^60^^,^^61^^,^^63^ with one combining group and individual sessions (Table 2),^59^ and one used an online intervention for individuals.^62^ Nine studies reported the number of sessions, which varied from eight^53^^,^^61^^,^^63^ to 16.^55^ In addition, two studies were regarded as involving ongoing interventions.^56^^,^^60^ Three studies included interventions delivered by a professional or by study personnel;^58^^,^^59^^,^^61^ the remaining six were delivered by teachers,^53^^–^^57^^,^^60^ by lay facilitators^63^ or online.^62^

Ten studies used self-report scales for assessing post-traumatic stress symptoms,^53^^–^^61^^,^^63^ and one used a self-report structured interview.^62^ Depression scores were derived using self-report scales in five studies,^54^^,^^56^^,^^57^^,^^60^^,^^61^ whereas a diagnostic interview was used in one.^62^ The two studies that reported anxiety measures both used self-report scales.^53^^,^^55^ Two studies reported follow-up post-traumatic stress symptom measures at 6 months and 12 months, respectively.^61^^,^^62^ Post-intervention depressive symptoms were reported by six studies,^54^^,^^56^^,^^57^^,^^60^^–^^62^ and two reported depressive symptoms at follow-up.^61^^,^^62^ Anxiety symptoms were reported post-intervention only (two studies).^53^^,^^55^

**Fig. 4:**
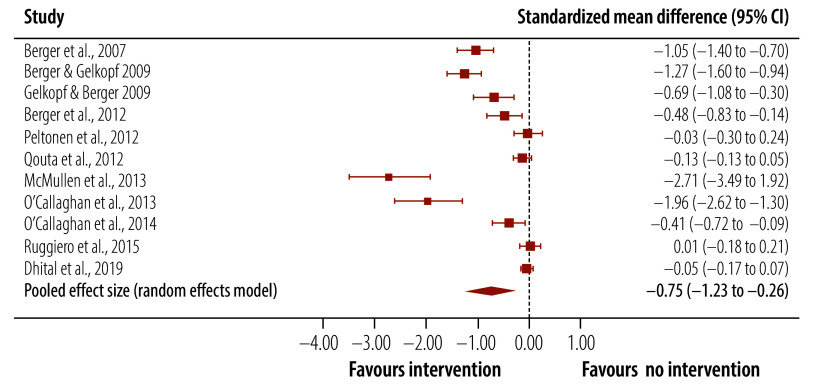
Effect of universal interventions on post-traumatic stress symptoms in children and adolescents in humanitarian crises, post-intervention assessments, 2002–2024

**Fig. 5:**
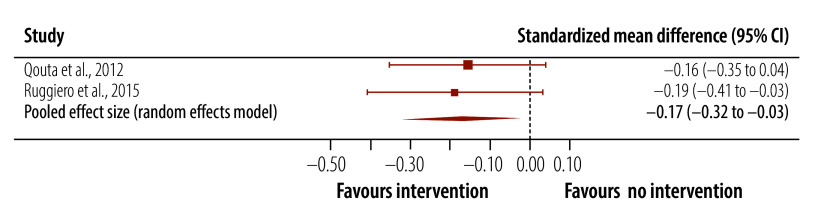
Effect of universal interventions on post-traumatic stress symptoms in children and adolescents in humanitarian crises, follow-up assessments, 2002–2024

The meta-analysis of trials of universal interventions demonstrated that post-traumatic stress symptom scores were significantly lower in children who received a universal psychosocial intervention compared to inactive controls (Table 4). The pooled effect size was medium and Hedges’ *g* was −0.75 (95% CI: −1.23 to −0.26; Fig. 4). For the two studies that reported post-traumatic stress symptoms at follow-up,^61^^,^^62^ the pooled effect size was small and Hedges’ *g* was −0.17 (95% CI: −0.32 to −0.03; Fig. 5). For depression scores, the effect size was not significant either post-intervention or at follow-up (Table 4). Nor was the effect size significant for anxiety scores post-intervention.

CI: confidence interval.

Note: the pooled effect size is expressed as Hedges’ *g* (95% CI).

CI: confidence interval.

Note: the pooled effect size is expressed as Hedges’ *g* (95% CI).

There was significant heterogeneity between studies. Funnel plots and an Egger’s test of funnel plot asymmetry indicated publication bias in the reported findings on post-traumatic stress symptoms post-intervention (online repository).^30^ However, a trim-and-fill procedure did not suggest there were any missing studies and, consequently, an adjusted effect size was not estimated.

Two studies reported CIs for the effect size that did not overlap with the CI of the pooled effect size.^58^^,^^59^ Sensitivity analyses that excluded these two studies found that the resulting effect size of universal interventions on post-traumatic stress symptoms was smaller but still significant (Hedges’ *g*: −0.44; 95% CI: −0.74 to −0.13).

A moderator analysis of the risk of bias was precluded because there was some concern of bias in 10 studies,^53^^–^^55^^,^^57^^–^^63^ and a high risk of bias in one (Table 2).^56^ In particular, there were some concerns about measurement of outcome and reporting bias in all studies because self-report assessments of outcome measures were generally used and there was no pre-specified plan to analyse outcomes. There were some concerns about the randomization process in three studies,^53^^,^^56^^,^^60^ and about missing data in one.^54^

A moderator analysis of the effect size of the interventions on post-traumatic stress symptoms post-intervention revealed no significant difference between human-made and natural disasters (Table 4). The effect size in natural disasters was small and not significant (Hedges’ *g*: −0.42; 95% CI −1.23 to 0.38). However, only three studies were included.^54^^,^^56^^,^^62^ No significant difference in effect size emerged between interventions delivered by specialists or non-specialists; however, the imbalance in study numbers made it difficult to draw a definitive conclusion.

An initial analysis indicated that the number of intervention sessions had a significant influence on effect size but the direction of the association was unclear. This finding appeared to be driven by two studies,^58^^,^^59^ each involving 15 sessions, that were identified as outliers. Once they were excluded, the number of sessions no longer showed a significant moderating impact. No significant difference in effect size was observed for country income. Nor was age found to be a significant moderating factor. However, the effect size for studies whose participants had a mean age under 13 years was smaller than that for studies with older participants and was not significant (Hedges’ *g*: −0.33; 95% CI: −0.70 to 0.04; Table 4).

## Discussion

Our meta-analysis evaluated the effectiveness of mental health and psychosocial support interventions for post-traumatic stress symptoms in children and adolescents across humanitarian settings. Initially we planned to review only targeted interventions; however, during our literature searches we encountered several studies of universal interventions that otherwise met our inclusion criteria. Due to their different aims, we conducted separate analyses of targeted and universal interventions. We included only randomized controlled trials because of their robust study design, and focused on studies with a passive control group to assess the overall utility of these interventions with the aim of supporting their use in humanitarian settings.

We did not directly compare targeted and universal interventions, but our meta-analysis found good evidence for the effectiveness of both, in keeping with previous reviews of studies conducted in humanitarian and low-income settings.^21^^–^^23^ The sustained benefits we found in both groups are important for children experiencing ongoing trauma in chronic humanitarian crises. However, only two studies of universal interventions reported follow-up assessments, which made it difficult to draw a meaningful conclusion. Nevertheless, given the evidence supporting universal interventions, there is a strong case for ensuring all children have access to mental health and psychosocial support rather than only those who meet specific diagnostic criteria, particularly where the extent of the trauma is overwhelming and likely to affect a large number of children. Nevertheless, targeted interventions may provide greater benefits where there is a smaller number of high-need cases.

Targeted interventions had a significant effect on depressive symptoms both post-intervention and at follow-up, though the effect size was small. This was not true of universal interventions. No significant effect on anxiety symptoms was found with either type of intervention. Other reviews of more generalized mental health and psychosocial support in similar settings have found them to be efficacious for treating a wide range of mental health outcomes,^21^ which suggests they may be of benefit when mental disorders other than PTSD are more prevalent.

In resource-limited settings, task-sharing is crucial for health-care provision. In keeping with previous literature,^22^ we found no significant difference in effect size between targeted interventions conducted by professional or lay facilitators. However, we were unable to make a meaningful comparison for universal interventions, for which further studies are needed. For resource-limited settings, it is important to be able to demonstrate that non-specialists and lay people can deliver interventions effectively as that would increase their accessibility and potential scalability. Additionally, we did not find a correlation between the number of intervention sessions and outcomes, which highlights the potential of shorter interventions where resources are scarce.

Most studies were conducted in group settings, which further suggests that the interventions are scalable. Although we identified too few studies involving individuals for a moderator analysis of group versus individual interventions, previous reviews support the use of group interventions for children with post-traumatic stress symptoms in humanitarian settings.^22^ Most studies we identified were based in schools, which remain a priority in humanitarian crises due to efforts to ensure children's access to education.^65^ It is encouraging that the interventions appear feasible in this setting, particularly where there is a need for rapid action.

 Additionally, the lack of long-term follow-up made it difficult to draw conclusions about the broader impact of early interventions on later child development. Moreover, only two studies of universal interventions reported follow-up measures. More generally, an inability to draw robust conclusions about the effect of universal interventions at follow-up is another important limitation of the extant literature.

The do-no-harm principle is a core concept in humanitarian work;^66^ although we did not identify any studies that demonstrated an overall harmful effect in any group, we could not exclude the possibility that there may have been a negative effect in some individuals.

Country income can serve as an accessible proxy for the availability of resources and the context of the intervention. Our moderator analysis found that the effectiveness of the interventions was not limited by country income.

The primary limitations of our meta-analysis relate to the individual study designs, which involved self-report measures with an inherent lack of blinding. The moderator analysis of targeted interventions indicated that the effect size was greater in studies with a high risk of bias, though substantial differences in effect size were also observed between other studies, albeit to a smaller extent. The high risk of bias we observed, particularly for studies of targeted interventions, may have affected the robustness of our conclusions and could have inflated the true effect size. In addition, three studies reported significant differences in post-traumatic stress symptoms between the intervention and control groups at baseline.^41^^,^^60^^,^^61^ However, as changes in symptoms from before to after treatment can be affected by spontaneous remissions in individuals exposed to trauma, the post-intervention effect size may provide a clearer indication of the benefit of the intervention than pre-intervention comparisons.

A further limitation of our study was the unequal distribution of studies between groups in the moderator analysis. For example, for both targeted and universal interventions, there were substantially more studies of human-made crises than natural disasters. Although we found no significant difference in the efficacy of the interventions between these two groups, the effect size for targeted interventions in natural disasters was not significant, perhaps because of the small pooled population, which highlights the need for further research in this area.

The high heterogeneity among studies we found in our analysis reflected: (i) the variability of the participants’ demographic characteristics, treatment modalities and symptom severity and the length of the intervention; (ii) the different challenges of natural and human-made disasters; and (iii) differences in outcome measures and when they were assessed. Additionally, variations in resource availability, which mirrored the diversity of humanitarian settings, probably had a large impact on how studies were conducted and affected the generalizability of their findings.

Although our study demonstrated that mental health and psychosocial support programmes are beneficial overall, we did not compare the relative efficacy of the different interventions identified. We found only nine studies that compared active interventions that met the criteria for our review,^67^^–^^75^ which suggests that further research is needed to identify the most effective interventions. However, the diversity of humanitarian settings makes comparisons challenging as the efficacy of an intervention can be influenced by its context. Nevertheless, we did identify instances in which several interventions were delivered in the same geographical region and crisis. It is possible that the availability of further studies could enable better comparisons to be made in such situations. However, conducting studies in unstable regions where the availability of resources fluctuates presents additional challenges and introduces confounding variables that make comparisons difficult.

Further research comparing the efficacy of interventions in similar settings will help policy-makers make evidence-based decisions about which programmes to implement. However, the choice of treatment modality in any context will be shaped by factors such as culture, logistics and resource availability. In addition, qualitative data on individual interventions and the specific challenges of implementing them in context would also benefit policy-makers.

Although our decision to include only randomized controlled trials in our analysis improved the robustness of our conclusions, it may have limited the comprehensiveness of the review because other study designs could have provided valuable insights into the real-world effectiveness of interventions in challenging settings.

The relatively small number of studies we identified highlights the need for further research. In particular, higher-quality randomized controlled trials should be conducted, though we acknowledge the difficulty of conducting research in unstable humanitarian settings. Future studies should address the limitations in study designs we identified. Although the absence of blinding is inherent to trials of psychological interventions, the strength of their conclusions could be improved by increasing the robustness of other aspects of study design, such as avoiding deviations from the intervention and using standardized outcome measures. An understanding of the sustained benefits of interventions is also crucial for confirming their effect in ongoing crises and for informing decision-making. However, collecting follow-up data can be challenging in unstable settings involving security risks and population displacement.

In conclusion, our analysis indicates that both targeted and universal psychological and psychosocial interventions are effective for treating post-traumatic stress symptoms in children and adolescents in humanitarian settings when compared to passive controls. Our findings highlight the value of mental health and psychosocial support programmes for addressing the mental health needs of children and adolescents experiencing trauma in such contexts. Future research should focus on conducting trials that have more robust study designs and that compare the effectiveness of different interventions in a range of contexts. Their findings will provide valuable evidence to guide decisions on resource allocation in humanitarian crises, thereby optimally serving the needs of the children and adolescents affected.
